# Investigating the Relationship between Smoking and Panic Disorder: A Cross-Sectional Study among US Adults

**DOI:** 10.1155/2024/8530368

**Published:** 2024-05-02

**Authors:** Arman Shafiee, Mana Goodarzi, Shahryar Rajai Firouzabadi, Ida Mohammadi, Dina Sadeghi, Mehrshad Zarei, Abolfazl Abdollahi, Ali Kolahdooz, Mahmood Bakhtiyari

**Affiliations:** ^1^Student Research Committee, School of Medicine, Alborz University of Medical Sciences, Karaj, Iran; ^2^Non-Communicable Diseases Research Center, Alborz University of Medical Sciences, Karaj, Iran; ^3^Shahid Beheshti University of Medical Sciences, Tehran, Iran; ^4^Student Research Committee, Isfahan University of Medical Sciences, Isfahan, Iran

## Abstract

**Background:**

Previous research on panic risk factors within the US population has been limited. This cross-sectional study is aimed at exploring the association between smoking and panic among adults in the United States.

**Methods:**

We conducted an analysis of data from the National Health and Nutrition Examination Survey.

**Results:**

The study included 2,222 participants. Those diagnosed with panic disorder were more likely to be female, unmarried, have lower income, engage in higher rates of smoking, and exhibit greater alcohol consumption. Participants who smoke cigarettes occasionally indicated a significant increase in panic disorder (unadjusted OR 95% CI: 4.396 [2.032-9.513]; *P* < 0.001). The significance of our results remained even after performing the multivariate analysis (full-adjusted OR 95% CI: 2.89 [1.30-6.42]). Furthermore, participants who never smoked cigarettes demonstrated strong and significantly low odds for panic disorder, regardless of adjustment (unadjusted OR 95% CI: 0.180 [0.055-0.591]). There was no association between pipe and cigar smoking and panic disorder in both unadjusted and full-adjusted models.

**Conclusion:**

This study highlights that smoking remains a significant risk factor for panic disorder, even after accounting for potential confounding variables. Further prospective longitudinal research should be done to investigate the causality between smoking and panic disorder.

## 1. Introduction

Panic disorder, characterized by recurrent and debilitating episodes of intense fear and apprehension, represents a significant mental health challenge affecting a substantial portion of the global population. While numerous factors have been explored in relation to the etiology and exacerbation of panic disorder, the association between cigarette smoking and this debilitating condition has emerged as a subject of paramount interest. This paper delves into an in-depth investigation of the intricate relationship between smoking behavior and panic disorder, drawing upon a critical review of existing literature and empirical evidence.

The relationship between cigarette smoking and panic disorder has been the subject of extensive research over the years. A growing body of evidence suggests that cigarette smoking may increase the risk of panic disorder [1–7], and quitting smoking may reduce such risk [1, 3].

In a critical review of the literature, we found that smoking cigarettes increases the risk of panic disorder with or without agoraphobia [1]. This association has been observed in both daily smokers and those who smoke persistently [3]. Moreover, studies have shown that smoking tends to precede the onset of panic and promote the development of the situation itself [1].

Findings from a prospective community study further support this association, revealing an increased risk for new-onset panic attacks with prior regular smoking and nicotine dependence [2]. Similarly, another study found that patients newly diagnosed with asthma who smoked during late childhood and/or adolescence had the greatest risk of developing panic disorder [8].

Interestingly, research has also shown a strong link between smoking and anxiety disorders during adolescence and early adulthood [9]. Heavy cigarette smoking during these stages has been associated with a higher risk of agoraphobia, anxiety, and panic disorders [9].

However, the relationship between smoking and panic disorder is complex and multifaceted. Some studies suggest that panic disorder may promote cigarette smoking as self-medication [1], indicating a potential bidirectional relationship. Furthermore, it has been suggested that shared vulnerabilities may promote both conditions [1].

While the exact nature of the relationship between cigarette smoking and panic disorder remains to be fully elucidated, current evidence points to a significant association between the two. This research is conducted to better understand this relationship and its underlying mechanisms.

## 2. Methods

### 2.1. Study Population

This study's primary objective is to examine the link between smoking and panic disorders in the adult population of the United States. We utilized data from the National Health and Nutrition Examination Survey (NHANES), which received approval from the National Center for Health Statistics (NCHS) Research Ethics Review Board for its data collection and definitions. Participants willingly joined the survey and received compensation along with a summary of the study's medical discoveries.

### 2.2. Data Collection

We utilized data from the initial three cycles of NHANES (1999-2004) since these cycles exclusively included the assessment of panic disorders. The evaluation process encompassed various examination components, including laboratory tests, medical assessments, and physiological measurements. To ensure the accuracy and quality of data collection, qualified medical professionals conducted these examinations employing state-of-the-art technology.

### 2.3. Definition of Variables

During the face-to-face interviews conducted in the mobile examination center (MEC) segment of the NHANES study, participants underwent a modified version of the World Health Organization Composite International Diagnostic Interview, Version 2.1 (CIDI-Auto 2.1). The CIDI is a standardized interview tool used to assess mental disorders and provide diagnoses based on the criteria outlined in the tenth revision of the International Classification of Diseases (ICD-10) and the fourth edition of the American Psychiatric Association's Diagnostic and Statistical Manual of Mental Disorders (DSM-IV). The NHANES CIDI, administered via computer, included three diagnostic modules focusing on conditions within the past 12 months, namely, panic disorder, generalized anxiety disorder, and depressive disorders. For this study, we derived our dependent variables from the final diagnostic tool for panic disorder (CIDPSCOR-Panic Score). To assess smoking cigarette status, we used NHANES data which asked participants if they had ever smoked 100 cigarettes in their lifetime. Additionally, we categorized smoking frequency based on recent smoking behavior into three groups: (1) frequent smokers, (2) occasional smokers, and (3) nonsmokers. Pipe and cigar smoking were defined as ever being used 20 times each in their lives. We selected covariates guided by relevant rationale and previously published research. We gathered demographic information, including respondents' age, gender, racial and ethnic background, educational level, marital status, annual family income (poverty index), alcohol consumption, and physical activity patterns, using standardized questionnaires.

### 2.4. Data Analysis

We conducted data analysis using StataCorp's Stata Statistical Software: Release 15. To accommodate the complex survey design, we utilized the full sample two-year MEC exam weight and masked variance pseudo-PSU for clustering. The entire dataset was weighted over a six-year period to ensure the representativeness of our findings, following the NHANES analytical guidelines formula. We employed multivariate and univariate logistic regression to investigate the relationship between smoking and panic disorders. The full-adjusted model was built by using those covariates with *P* value < 0.2 in the univariate analysis. Results with a *P* value less than 0.05 were considered statistically significant.

## 3. Results

### 3.1. Baseline Characteristics

Table 1 shows the baseline characteristics of the included population. We found that some characteristics were more prevalent among participants with a diagnosis of panic disorder, including the female gender, not being married, alcohol consumption, smoking, and a higher frequency of smoking. We did not find age, ethnicity, education, smoking pipe, smoking cigars, or physical activity to be markedly different among the populations with and without a diagnosis of panic disorder.

### 3.2. Association between Smoking and Panic Disorder

Table 2 delineates the unadjusted ORs from the logistic regression models evaluating the associations between covariates and the diagnosis of panic disorder. The age of the participants or years of education did not turn out to yield a statistically significant OR in relation to panic disorder in any of the models. On the other hand, poverty, marital status, alcohol use, and the female gender were associated with panic disorder. Participants who smoke cigarettes occasionally indicated a significant increase in panic disorder (unadjusted OR 95% CI: 4.396 [2.032-9.513]; *P* < 0.001). The significance of our results remained even after performing the multivariate analysis (model 1 OR 95% CI: 4.65 [2.09-10.37]; model 2 OR 95% CI: 2.89 [1.30-6.42]). Furthermore, participants who never smoked cigarettes demonstrated strong and significantly low odds for panic disorder, regardless of adjustment (unadjusted OR 95% CI: 0.180 [0.055-0.591]). There was no association between pipe and cigar smoking and panic disorder in both unadjusted and full-adjusted models (Table 3).

## 4. Discussion

Our representative cross-sectional study attempted to elucidate the relationship between smoking and panic disorder using a reliable sample from the NHANES database. After univariate analysis, smoking cigarettes and frequency both showed a correlation with the incidence of panic disorder, with smokers being more susceptible to panic disorder and those who smoke occasionally vs. frequently being less susceptible. Those who do not smoke also showed significantly lower odds of developing panic disorder in comparison to frequent smokers.

Our study shows that smoking is related to the development of panic disorder, regardless of frequency. This connection has been noted in previous works as well [1–5, 7, 9], with various biological mechanisms being proposed to elaborate the link between panic disorder and smoking. Even though the pathophysiology of panic is not well understood, there is abundant evidence that panic is associated with noradrenergic abnormalities [10–12].

The nicotine found in cigarettes has pronounced adrenergic effects such as increasing heart rate and blood pressure, both of which are associated with panic disorder [13–15]. Nicotine also crosses the blood-brain barrier (BBB) and activates several pathways in the central nervous system which causes the release of norepinephrine and serotonin [16, 17], both of which have been implicated in the pathogenesis of panic disorder [18, 19] and are targeted when treating it [20]. Smoking can also cause lung impairments which mimic panic symptoms [21]. On the other hand, there are several studies considering smoking as a self-medication for anxiety spectrum disorders, such as panic [22–24].

Our findings are consistent with the results of Fargamfar et al. [5]. In their study, it was found that smoking increases the incidence of panic attacks among psychiatric patients. Unlike our study, they conducted a case-control study, and patients were divided into 2 groups: those who already had or had a history of panic attacks were in the case group, and those who did not have panic attacks in the past year were in the control group. Their study however was limited by a small sample size.

In a similar study, Breslau and Klein [4] showed that daily smoking greatly increases the first occurrence of panic attacks and disorders. They also reported that those who continued to smoke were at higher risk of panic attacks compared to those who quit smoking.

Similarly, Bakhshaie et al. [3] conducted a study among adults in mid-adulthood in the United States from 1994 to 2005 which showed that smoking leads to an increasing likelihood of the onset and endurance of panic attacks. Furthermore, it indicated that smoking cessation assists in reducing this risk, although they suggested testing several clinical trials to prove this hypothesis.

Another study by Johnson et al. [9] demonstrated a strong positive relationship between smoking during adolescence and panic disorder in adulthood. However, this connection faded in early adulthood in their study. The effect of age was studied in our work as well, yet we found no connection in univariate analysis, as well as in our adjusted models, and the association between smoking and panic disorder remained valid after adjustment for age. This conflict however should be investigated further as our database lacks a wide spectrum of age groups.

Retrospective cohort research among young adults by Isensee et al. [2] illustrated the relationship between preceding smoking and an increased risk for panic attacks and panic disorders. However, they could not rule out the reverse pathway. Additionally, it showed that the association between panic and smoking, unlike other anxiety disorders, is not influenced by comorbidities. This study also divided smokers into groups according to the number of years of regular smoking and the number of cigarettes per year, whereas we divided smokers into three main groups based on their interviews.

A recent cohort study by Wu et al. [7] showed the association between asthma and early smoking with panic disorder in young adults. They categorized approximately 162000 participants into two asthma and nonasthma groups, and based on their results, patients who were diagnosed with asthma had a higher risk of incident panic disorder, and specifically, those who smoked during late childhood or adolescence had the greatest risk of getting the panic disorder. We did a cross-sectional study with a smaller sample size, while Wu et al. did a mop cohort-based study with a great sample size. Moreover, our study was gathered through a combination of interviews and physical examinations, whereas Wu et al. used a regional sample of southern Taiwan.

In our study, panic disorder is reported more frequently in females. Panic disorder is not gender-specific, but research has shown different prevalence rates between men and women [25, 26]. Studies show that women are more likely to be diagnosed with panic disorder due to a combination of psychological and sociocultural factors [27]. In accordance with our study, Kessler et al., based on the National Comorbidity Survey, indicated that the prevalence of panic is higher among women compared to men [25].

Some studies show that people with lower incomes may be at higher risk of panic disorder. Conditions such as financial instability and limited access to health care may contribute to the development of anxiety disorders, namely, panic disorder [28–30]. Dijkstra-Kersten et al. considered the impact of income and financial strains on the presence of depression and anxiety disorder in a 4-year follow-up study among 1250 participants. Consistent with our results, they concluded that people with mild or severe financial strains are more likely to develop depression and anxiety disorders. However, they could not consider the change of financial strains during the 4-year follow-up which may impress the onset and recurrence of depression and anxiety disorder. Also, they could not establish the exact association between income difficulties and the onset and recurrence of anxiety disorder [29].

The link between anxiety and smoking is multifaceted, with each condition potentially exacerbating the other [31]. Many individuals with anxiety may smoke to self-medicate, seeking temporary relief from nicotine, despite its fleeting effects [32]. Conversely, nicotine withdrawal can heighten anxiety, creating a challenging cycle for those attempting to quit smoking. This bidirectional relationship is influenced by biological factors, such as nicotine's impact on neurotransmitter systems like dopamine and serotonin, which regulate mood and anxiety [33]. Additionally, social and environmental stressors can intensify the reliance on smoking as a coping mechanism. The progression from anxiety to panic disorder involves the escalation of anxiety symptoms to acute, intense episodes of panic attacks, marked by overwhelming fear and physical discomfort. This transition can be facilitated by several factors in the context of smoking [34]. The sensitization to nicotine withdrawal symptoms, which mimic those of panic attacks, can heighten anxiety about these sensations, potentially leading to panic disorder [35]. Chronic smoking may also induce neurobiological changes that increase susceptibility to panic attacks, while psychological reliance on smoking for anxiety management can exacerbate feelings of helplessness and fear, key components of panic disorder [36].

The current study has several strengths; the gathered data from the NHANES database makes our findings more reliable in comparison to other works. Our study also benefits from access to details of cofounding factors, allowing us to study the relationship between smoking and panic disorder in a similar style to cohort studies. Also, there are several limitations to this study. First, our survey is a cross-sectional study focusing on the data from the NHANES database, and it is only a small sample of a large and ethnic study population; therefore, our results are not necessarily applicable to other populations than US adults. Second, this study cannot make a causal inference between smoking and panic disorder due to its method. Third, unlike cohort and case-control studies, there are no prospective or retrospective follow-ups; thus, it is hard to interpret the relationship between smoking as an exposure and panic disorder as an outcome, and the critical effect of smoking cessation on the risk of panic disorder could not be studied. Further prospective longitudinal research should be done to investigate the causality. Moreover, self-reported data can prompt recall bias. Participants may not give an accurate record of the number of cigarettes they smoke per year. Also, habits like smoking tend to be underreported. Additionally, the data we analyzed were from US citizens; therefore, future studies should aim to replicate results in a larger target population including other origins and countries. Lastly, since there was no newer data after 2004 in the NHANES study, our results are limited to those participants from 1999 to 2004. We reported data on the association between pipe and cigar smoking and panic disorder; however, recently, newcomers to tobacco smoking methods such as electronic cigarettes and vaping have been replaced with old methods of using nicotine [37]. Studies are needed to assess the effect of these new technologies on panic disorder.

In conclusion, the current study revealed that smoking may increase the risk of panic disorder, even after adjustment for confounding factors.

## Figures and Tables

**Table 1:** Table of characteristics.

Variable	Diagnosed with panic disorder		No diagnosis of panic disorder		
	Frequency	95% CI	Frequency	95% CI	*P* value
Age^a^	30.29	[28.16-32.43]	29.66	[29.29-30.02]	
Gender					
Male	0.018	[0.011-0.0292]	0.982	[0.9708-0.989]	0.0392
Female	0.0346	[0.023-0.0516]	0.9654	[0.9484-0.977]	
Total	0.0264	[0.0192-0.0363]	0.9736	[0.9637-0.9808]	
Ethnicity/race					
Non-Hispanic White	0.0276	[0.0184-0.0412]	0.9724	[0.9588-0.9816]	0.8464
Non-Hispanic Black	0.0234	[0.0119-0.0456]	0.9766	[0.9544-0.9881]	
Hispanic	0.0228	[0.01-0.0509]	0.9772	[0.9491-0.99]	
Total	0.0261	[0.0189-0.036]	0.9739	[0.964-0.9811]	
Education					
Less than 12 years	0.0293	[0.0186-0.0459]	0.9742	[0.9619-0.9826]	0.6862
More than 12 years	0.0258	[0.0174-0.0381]	0.9707	[0.9541-0.9814]	
Total	0.0265	[0.0192-0.0364]	0.9735	[0.9636-0.9808]	
Marital status					
Married	0.0166	[0.0104-0.0265]	0.9834	[0.9735-0.9896]	0.0077
Not married	0.0376	[0.0247-0.057]	0.9624	[0.943-0.9753]	
Total	0.0257	[0.0184-0.0357]	0.9743	[0.9643-0.9816]	
Income status					
Above poverty	0.0206	[0.0137-0.031]	0.9794	[0.969-0.9863]	0.008
Below poverty	0.0479	[0.0285-0.0794]	0.9521	[0.9206-0.9715]	
Total	0.0251	[0.018-0.035]	0.9749	[0.965-0.982]	
Alcohol consumption					
Yes	0.0511	[0.0274-0.0933]	0.9489	[0.9067-0.9726]	0.0338
No	0.0239	[0.0165-0.0347]	0.9761	[0.9653-0.9835]	
Total	0.0281	[0.0203-0.0389]	0.9719	[0.9611-0.9797]	
Smoking cigarettes					
Yes	0.046	[0.0317-0.0662]	0.954	[0.9338, 0.9683]	0.0001
No	0.0108	[0.0056-0.0209]	0.9892	[0.9791, 0.9944]	
Total	0.0265	[0.0192-0.0363]	0.9735	[0.9637, 0.9808]	
Smoking cigarettes frequency					
Frequently smokes	0.071	[0.0481-0.1036]	0.929	[0.8964-0.9519]	0.0011
Occasionally smokes	0.0206	[0.0067-0.0612]	0.9794	[0.9388-0.9933]	
Never smokes	0.0136	[0.0043-0.042]	0.9864	[0.958-0.9957]	
Total	0.046	[0.0317-0.0662]	0.954	[0.9338-0.9683]	
Smoking pipe					
Yes	0.0591	[0.0179-0.1776]	0.9409	[0.8224-0.9821]	0.16
No	0.0254	[0.0182-0.0354]	0.9746	[0.9646-0.9818]	
Total	0.0265	[0.0192-0.0363]	0.9735	[0.9637-0.9808]	
Smoking cigar					
Yes	0.0206	[0.0078-0.0533]	0.9794	[0.9467-0.9922]	0.58
No	0.0272	[0.0195-0.038]	0.9728	[0.962-0.9805]	
Total	0.0265	[0.0192-0.0363]	0.9735	[0.9637-0.9808]	
Physically active					
No	0.0278	[0.0188-0.0409]	0.9722	[0.9591-0.9812]	0.6611
Yes	0.023	[0.0111-0.0469]	0.977	[0.9531-0.9889]	
Total	0.0265	[0.0193-0.0364]	0.9735	[0.9636-0.9807]	

^a^Data is presented as mean. Abbreviation: CI: confidence interval.

**Table 2:** OR of different variables associated with panic disorder.

Variable	Unadjusted OR (95% CI)	*P* value
Age^a^	1.019 [0.956-1.086]	>0.05
Smoking cigarettes		
No	Reference	
Yes	4.396 [2.032-9.513]	<0.001
Smoking cigarettes frequency		
Frequently smokes	Reference	
Occasionally smokes	0.275 [0.084-0.902]	<0.05
Never smokes	0.180 [0.055-0.591]	<0.05
Smoking pipe		
No	Reference	
Yes	2.41 [0.67-8.67]	0.17
Smoking cigar		
No	Reference	
Yes	0.75 [0.27-2.12]	0.58
Alcohol consumption		
No	Reference	
Yes	2.196 [1.046-4.609]	<0.05
Gender		
Female	Reference	
Male	0.512 [0.269-0.977]	<0.05
Ethnicity/race		
Non-Hispanic White	Reference	
Non-Hispanic Black	0.844 [0.365-1.953]	>0.05
Hispanic	0.822 [0.328-2.064]	>0.05
Education		
Less than 12 years	Reference	
More than 12 years	0.879 [0.464-1.665]	>0.05
Marital status		
Not married	Reference	
Married	0.433 [0.233-0.805]	<0.05
Income status		
Below poverty	Reference	
Above poverty	0.419 [0.218-0.803]	<0.05
Physically active		
No	Reference	
Yes	0.823 [0.336-2.011]	>0.05

^a^Each 1 year increase in age.

**Table 3:** Adjusted OR of the relationship between smoking and panic disorder.

Outcomes	Model 1^a^ OR (95% CI)	*P* value	Model 2^b^ OR (95% CI)	*P* value
Smoking cigarettes				
No	Reference		Reference	
Yes	4.65 [2.09-10.37]	*P* < 0.001	2.89 [1.30-6.42]	*P* < 0.01
Smoking cigarettes frequency				
Frequently smokes	Reference		Reference	
Occasionally smokes	0.28 [0.09-0.92]	*P* < 0.05	0.37 [0.11-1.15]	*P* = 0.85
Never smokes	0.16 [0.05-0.53]	*P* < 0.01	0.30 [0.09-1.00]	*P* = 0.05
Smoking pipe				
No	Reference		Reference	
Yes	3.50 [0.96-10.73]	*P* = 0.056	1.66 [0.34-8.08]	*P* = 0.57
Smoking cigar				
No	Reference		Reference	
Yes	1.09 [0.35-3.40]	*P* = 0.87	0.30 [0.07-1.24]	*P* = 0.10

^a^Adjusted for age and sex. ^b^Further adjusted for age, sex, and covariates with *P* value < 0.2 in univariate analysis.

## Data Availability

The data will be available upon reasonable request from the corresponding author.
